# Supplementary figures and images for: Cannabis in Hematology Survey Study (CHESS): A Longitudinal Investigation on Uses, Attitudes, and Outcomes of Cannabis Among Hematology Patients Undergoing Hematopoietic Stem Cell Transplant
Source: Int J Environ Res Public Health. 2025 Jun 23;22(7):990. doi: 10.3390/ijerph22070990 (PMC12294567; doi:10.3390/ijerph22070990)

**Figure S1.** Highlighted pre and post treatment comparisons.

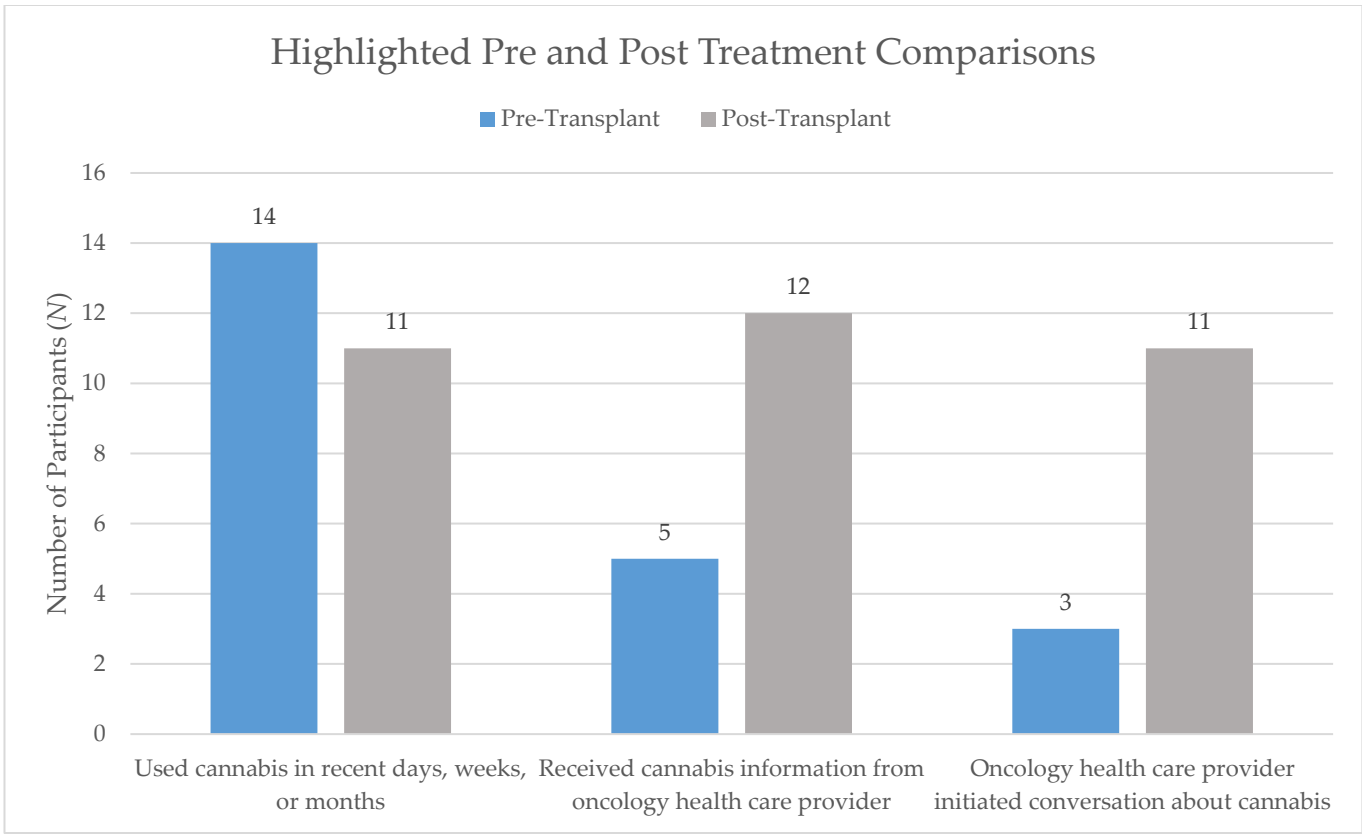

Supplement: Supplementary file 1 [file ijerph-22-00990-s001.zip › ijerph-3592971-supplementary.pdf]
